# Development of a Gene-Based Soybean-Origin Discrimination Method Using Allele-Specific Polymerase Chain Reaction

**DOI:** 10.3390/foods12244497

**Published:** 2023-12-16

**Authors:** Kie-Chul Jung, Bo-Young Kim, Myoung-Jin Kim, Nam-Kuk Kim, Jihun Kang, Yul-Ho Kim, Hyang-Mi Park, Han-Sub Jang, Hee-Chang Shin, Tae-Jip Kim

**Affiliations:** 1 Experiment & Research Institute, National Agricultural Products Quality Management Service, Gimcheon 39660, Republic of Korea; kcjung@korea.kr (K.-C.J.); kby720@korea.kr (B.-Y.K.); gumdung3@korea.kr (M.-J.K.); nkvirus@korea.kr (N.-K.K.); jjhs@korea.kr (H.-S.J.); 2 Division of Animal, Horticultural and Food Sciences, Graduate School of Chungbuk National University, Cheongju 28644, Republic of Korea; kimcholl@naver.com; 3 National Institute of Crop Science, Rural Development Administration, Suwon 16429, Republic of Korea; kimyuh77@korea.kr (Y.-H.K.); parkhm2002@korea.kr (H.-M.P.)

**Keywords:** soybean, whole-genome sequencing, single-nucleotide polymorphism, origin discrimination, genetic markers

## Abstract

A low soybean self-sufficiency rate in South Korea has caused a high import dependence and considerable price variation between domestic and foreign soybeans, causing the false labeling of foreign soybeans as domestic. Conventional soybean origin discrimination methods prevent a single-grain analysis and rely on the presence or absence of several compounds or concentration differences. This limits the origin discrimination of mixed samples, demonstrating the need for a method that analyzes individual grains. Therefore, we developed a method for origin discrimination using genetic analysis. The whole-genome sequencing data of the Williams 82 reference cultivar and 15 soybean varieties cultivated in South Korea were analyzed to identify the dense variation blocks (dVBs) with a high single-nucleotide polymorphism density. The PCR primers were prepared and validated for the insertion–deletion (InDel) sequences of the dVBs to discriminate each soybean variety. Our method effectively discriminated domestic and foreign soybean varieties, eliminating their false labeling.

## 1. Introduction

Soybean is an important food ingredient and a primary source of nutrients worldwide, with an abundance of isoflavones, carbohydrates, fats, and proteins. Soybean has been utilized mainly in the production of vegetable oil and as an ingredient in fermented foods, such as soybean paste, soy sauce, red pepper paste, fermented soybean paste, and fermented whole soybean. It has also been consumed in the form of processed foods, including soybean milk and soybean curd. Owing to its enrichment with the essential amino acid lysine, soybean is the main protein source in countries that consume rice as a staple food [1].

The soybean self-sufficiency rate in South Korea is ≤30%, resulting in a high import rate. The main producers of soybeans are the United States of America (U.S.), Brazil, Argentina, India, and China. Thus, South Korea primarily imports soybeans from the U.S., Canada, and China. The wholesale price of imported soybeans is considerably low, at 25% of the price of domestic soybeans [2].

Owing to the substantial price difference between domestic and imported soybeans, sellers might falsely label the country of origin to earn unfair profits. In 2022, the National Agricultural Products Quality Management Service (NAQS) in South Korea announced that soybean curd ranked sixth and soybean ranked seventh among the 156 items violating the Act on Origin Labeling of Agricultural and Fishery Products [3].

The origin of soybean is currently discriminated based on the difference in inorganic compounds using energy dispersive X-ray fluorescence spectrometry (ED-XRF). Furthermore, the absorbance difference across organic compounds is determined using Fourier transform near-infrared spectroscopy (FT-NIRS) [4,5,6]. The conventional physicochemical method of analysis enables a markedly simple and rapid analysis that requires no pretreatment besides the grinding of samples. However, a single grain of soybean cannot be analyzed. Additionally, the minimum sample mass required for analysis is 5–50 g. These limitations could pose difficulties in the origin discrimination of mixed samples.

Extensive research has been conducted to distinguish growing plant varieties through morphological classification. Experts in origin determination have traditionally relied on distinguishing varieties based on seed shape, gloss, and color. However, a challenge arises as results tend to vary depending on the evaluator’s level of expertise and experience. To address this issue, the field has turned to DNA molecular marker technology for breed identification [7].

The most frequently used method in soybean variety analysis is the simple sequence repeat (SSR), or microsatellite, approach used to establish barcode systems. However, this method requires expensive equipment such as a DNA sequencer or chip electrophoresis and a large number of markers, making it difficult to adopt as a general laboratory method [8,9]. The second most used PCR method is the cleaved amplified polymorphic sequence (CAPS) with a single-nucleotide polymorphisms (SNPs) approach. This method is time-consuming owing to the involvement of restriction enzymes in addition to PCR [10]. Recently, insertion–deletion (InDel) markers have achieved high reproducibility and are recognized as efficient molecular markers for distinguishing cultivars based on codominance [11]. In particular, these markers are attracting attention from researchers because they are relatively simple compared to other molecular markers. Soybeans possess 20 chromosomes with a known genome euchromatic DNA size of 705 Mb. These chromosomes are categorized into sVB (sparse variation block), characterized by the absence of chromosomal mutation, and dVB (dense variation block), which occurs infrequently. Notably, studies have indicated the presence of dVB within 100 kb of the chromosome, and that genetic recombination does not occur often during the breeding process compared to the linkage disequilibrium block of 90–574 kb in soybean varieties [12]. In a previous study, a method was proposed to differentiate between dVB and sVB, focusing on the identification of InDel markers within dVB to develop soybean variety identification methods [13,14]. Some studies have proposed a DNA barcode method for discriminating 147 soybean varieties using the genomic DNA extracted from soybean leaves by using a selected set of InDel markers of dense variation blocks (dVB). However, this method focuses on domestic soybean varieties, and there is a limitation in identifying genetic diversity patterns of the imported varieties [15]. Therefore, this study aimed to utilize genetic analysis to discriminate the varieties of soybean cultivated in South Korea. Unlike the physicochemical analysis techniques known to date, the gene-based analysis technology developed in this study can analyze each individual grain, thereby ensuring a high identification accuracy. Therefore, it is likely that a quantitative analysis will be able to identify the origin of mixed samples. The proposed origin discrimination table could eliminate the false labeling of foreign soybean varieties as domestic ones, thereby reducing unethical price markups by sellers.

## 2. Materials and Methods

### 2.1. Sample Collection

The 16 soybean varieties used as standard samples (15 Korean varieties and 1 American variety) were obtained from the Rural Development Administration. A total of 1096 samples (630 domestic and 466 imported) were collected from soybean farms, ports of entry, and large distributors between 2019 and 2021. The collected samples were used to construct the origin judgment value database. Furthermore, 60 soybean samples (30 domestic and 30 imported) were collected in 2022 to validate the origin discrimination table. The 30 domestic soybeans were obtained from 27 farms and local food markets nationwide. In addition, the imported soybeans included 11, 10, 4, 3, and 2 from the U.S., China, Canada, Thailand, and Vietnam, respectively. The collected samples were stored in a −20 °C freezer.

### 2.2. Genomic DNA Extraction

For DNA extraction from embryos of collected soybean samples, we used the Magnetic Bead System of an automatic nucleic acid extraction device (Hamilton Microlab Star^®^, Hamilton Co., Reno, NV, USA). The NanoDrop 2000 spectrophotometer (Thermo Fisher Scientific, Waltham, MA, USA) was used to measure the concentration and purity of the extracted genomic DNA. Finally, the purity was validated at 260/230 nm and 260/280 nm before PCR analysis to ensure that it fell within the range of 1.8–2.0.

### 2.3. Selection of Test Varieties and Molecular Markers

The complete whole-genome sequencing data of the soybean Williams 82 cultivar reference genome and those of the 15 known domestic varieties (Daewon, Taekwang, Pungsannamul, Seonyu, Daepung, Sinhwa, Hwangkeum, Nampung, Cheonsang, Uram, Hwangkeumol, Saedanbaek, Pungwon, Cheongja1, and Cheongja3) were analyzed to compare dVB with a high SNP density. Based on the result, 11 InDel markers were selected to identify 16 standard samples.

### 2.4. Primer Preparation and PCR Analysis

**Figure 1:**
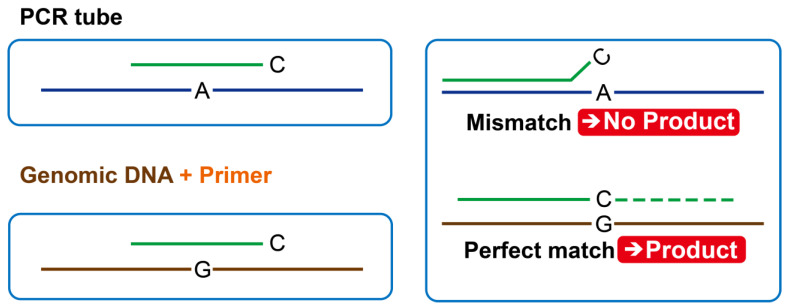
Confirmation of genetic patterns using allele-specific polymerase chain reaction (PCR) markers. The letters G, C, and A represent nucleotides.

The 11 selected InDel markers were used to prepare the primers via allele-specific PCR to allow for easy differentiation based on the state of PCR amplification (Figure 1). For the PCR mixture, 40 ng of genomic DNA, 0.2 pmol of primer, and 10 μL of anti-HS Taq Premix (2× reaction buffer, 4 mM MgCl_2_, 0.5 mM dNTP, and 1 unit of Anti HS Taq DNA polymerase (TNT Research, Jeonju-si, South Korea)) were used to perform 10 min pre-denaturation at 94 °C, 30 s denaturation at 94 °C, 30 s annealing at 56 °C, and 10 min extension at 72 °C. The PCR reaction was terminated at 4 °C. Afterward, the amplified PCR product was loaded to 3% agarose gel with GoldView (SBS Genentech, Beijing, China) for 30 min electrophoresis at 200 V. The PCR product was confirmed using ultraviolet light. Furthermore, confirmation of the PCR product was carried out through QIAxcel electrophoresis (QIAGEN, Hilden, Germany).

### 2.5. Determination of Judgment Values

Marker 1 is a specific marker for soybean’s endogenous genes, enabling the determination of whether the analyzed sample is soybean or not. In essence, successful amplification in PCR is essential for discerning the origin of soybeans. Therefore, marker 1 was exceptionally assigned a score of 1. Subsequently, markers 2 to 11 were scored as 2^n^ with each marker obtaining the following points through allele-specific PCR amplification: marker 2 (2 points), marker 3 (4 points), marker 4 (8 points), marker 5 (16 points), marker 6 (32 points), marker 7 (64 points), marker 8 (128 points), marker 9 (256 points), marker 10 (512 points), and marker 11 (1024 points). The cumulative sum of scores from all amplified markers was then calculated to determine the overall judgment value. The calculated scores using 11 markers could theoretically allow for the genetic diversity pattern discrimination of 2048 species based on judgment values. After checking the genetic polymorphism of the 16 standard samples, 1096 collected soybean samples were used to generate the origin discrimination tables.

### 2.6. Validation of the Origin Discrimination Table

The origin discrimination formula was validated based on judgment values using the 11 markers applied to 30 domestic and 30 imported soybeans. For the discrimination formula, the sensitivity, selectivity, and efficiency were estimated through qualitative analysis [16,17].

(1) $$Sensitivity=\left(\frac{TD}{TD\;+\;FD}\right)\times 100$$

(2) $$Selectivity=\left(\frac{TF}{TF\;+\;FF}\right)\times 100$$

(3) $$Efficiency=\left(\frac{TD\;+\;TF}{TD\;+\;FD\;+\;TE\;+\;FF}\right)\times 100$$

True Domestic Product (TD) indicates a domestic sample identified as domestic based on the discrimination result. False Domestic Product (FD) indicates an imported sample identified as domestic based on the discrimination result. True Foreign Product (TF) indicates an imported sample identified as foreign based on the discrimination result. False Foreign Product (FF) indicates a domestic sample identified as foreign based on the discrimination result. Sensitivity indicates the level at which the discrimination table can correctly identify a domestic sample. Similarly, selectivity indicates the level at which the discrimination table can correctly identify a foreign sample. To evaluate the prediction performance of the established discrimination table, the judgment values of the tested samples were applied to the table. The percentage of the discrimination of domestic samples as domestic was set as the domestic predictive rate and that of the discrimination of foreign samples as foreign was set as the foreign predictive rate.

## 3. Results and Discussion

### 3.1. Validation of the Selected Molecular Markers

Seventeen molecular markers were selected from the InDel region of dVB that was predicted to enable the identification of the 16 soybean varieties of standard samples through biodata analyses. The PCR amplification of the selected markers was checked against the Williams 82 reference. However, certain markers led to nonspecific reaction products unable to be used in allele-specific PCR, which relies on the presence or absence of PCR-amplified products for molecular markers. Ultimately, 11 molecular markers capable of allele-specific PCR were selected through verification, and these were used in tests to determine the origin discrimination (Table 1).

### 3.2. Multiplex Allele-Specific PCR Analysis

**Figure 2:**
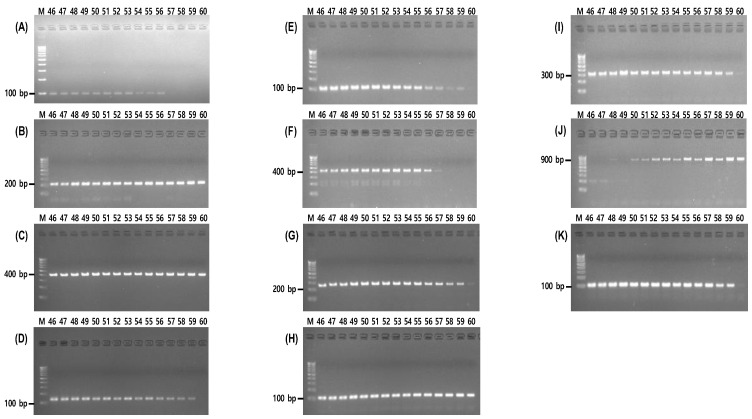
Polymerase chain reaction amplification of 11 markers for soybean origin discrimination. (**A**) Marker 1 (102 bp), (**B**) marker 2 (238 bp), (**C**) marker 3 (473 bp), (**D**) marker 4 (138 bp), (**E**) marker 5 (107 bp), (**F**) marker 6 (459 bp), (**G**) marker 7 (246 bp), (**H**) marker 8 (112 bp), (**I**) marker 9 (324 bp), (**J**) marker 10 (872 bp), (**K**) marker 11 (112 bp).

**Figure 3:**
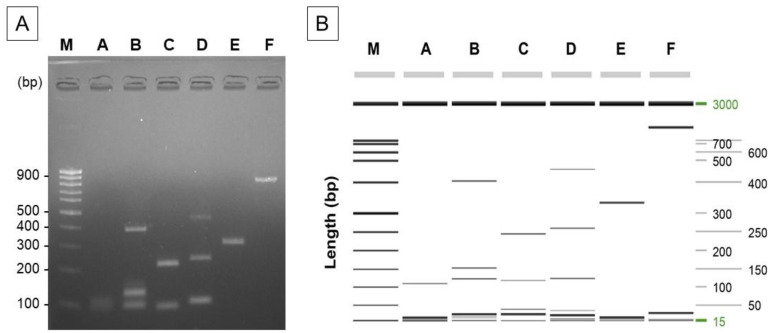
Simultaneous detection performance and specificity of multiplex PCR based on 11 InDel markers for soybean origin identification by (**A**) agarose gel electrophoresis and (**B**) capillary electrophoresis. Lane M: (**A**) 100 bp DNA ladder (ST-M100, TNT Research, Korea), (**B**) 15 bp to 3 kb DNA ladder (QIAxcel DNA Fast Analysis kit, QIAGEN, Hilden, Germany); lane A: marker 1 (102 bp); lane B: marker 3 (473 bp), marker 4 (138 bp), marker 11 (112 bp); lane C: marker 2 (238 bp), marker 5 (107 bp); lane D: marker 6 (459 bp), marker 7 (246 bp), marker 8 (112 bp); lane E: marker 9 (324 bp); lane F: marker 10 (872 bp).

In previous studies, the interpretation of results obtained via PCR was complicated as both the presence or absence of amplification of the InDel marker and the size difference in the PCR amplification products were considered. In this study, the allele-specific PCR including the InDel sequences allowed for an intuitive result interpretation based on the state of PCR amplification. Although the results can be confirmed more easily than with existing analysis methods, it still takes much time and labor to confirm the origin of one sample because, to do this, PCR must be performed on 24 single soybean grains and confirmed via electrophoresis. To solve this problem, we set up six groups of 11 markers to enable multiplex PCR. To validate the feasibility of allele-specific PCR for InDel markers 1 to 11 at a single annealing temperature, amplification was assessed across a temperature range of 46 to 60 °C. Among these temperatures, we found that only the target product was amplified at 56 °C, without any non-specific PCR products (Figure 2). We attempted to confirm 11 InDel markers via a single multiplex PCR; however, because of the size of the amplified product and non-specific reaction, we optimized a total of six groups (Figure 3). Accordingly, we were able to significantly reduce the time to determine the geographical origin through multiplex allele-specific PCR.

### 3.3. Determination of Judgment Values for Standard and Test Samples

Because a minimum sample of 24 single grains is required for 95% reliability, 200 g of the collected sample was evenly distributed with a grain spreader, and 24 single grains were ultimately sampled [18]. After DNA extraction, multiplex PCR analysis was performed on the samples using the 11 InDel markers that were assigned unique scores. Then, the scores given to the amplified markers were calculated to confirm the judgment values.

First, we confirmed, through analysis, whether the 16 standard samples were clearly identified. Among the 16 varieties (15 domestic and 1 imported), 14 varieties (13 domestic and 1 imported) could be discriminated (Table 2). The two domestic varieties that could not be discriminated were Seonyu and Hwangkeumol, which shared an identical judgment value (655).

Second, to set the judgment value for a variety of soybean samples, 1096 soybean samples were collected to include the domestic soybeans cultivated in South Korea, those imported through the port of entry, and the foreign soybeans currently being distributed. As a result, domestic and imported soybean varieties could be classified based on 53 and 70 judgment values, respectively (Table 3 and Table 4). These varieties had four overlapping judgment values (671; 1183; 1215; and 1695) that prevented origin discrimination across the corresponding domestic and imported soybeans. 

**Figure 4:**
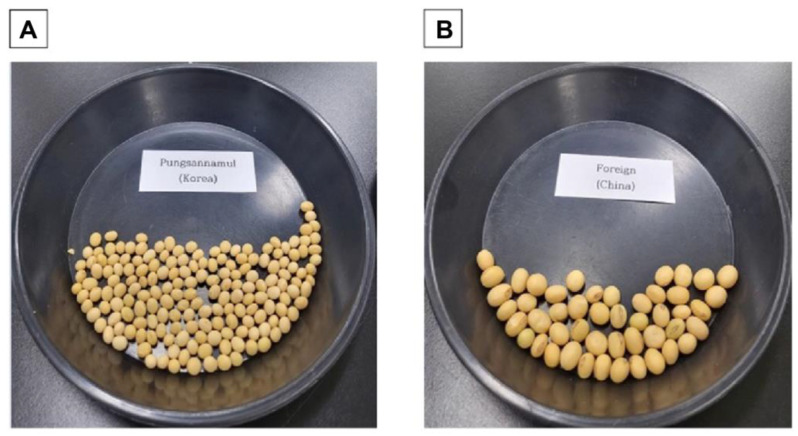
Morphological comparison between Pungsannamul and Chinese varieties with an overlapping judgment value (1215). Morphological comparison between (**A**) Pungsannamul and (**B**) Chinese varieties with an overlapping judgment value (1215).

The aim of this study was to discriminate the origin of soybeans based on the variation of judgment values between domestic and imported varieties rather than accurately identify the variety of soybeans. For the two domestic varieties that could not be discriminated in the standard samples, the judgment value (655) did not overlap with the judgment values of imported soybeans. The four judgment values that were the same for domestic and foreign products accounted for approximately 3.4% of the total judgment values, and the samples for which judgment was impossible accounted for approximately 5% of the total sample. However, if foreign varieties (1215) with the same judgment value as Pungsannamul (domestic varieties) are excluded because they have obvious morphological differences (Figure 4), the rate of the inability to make a judgment decreases from approximately 3.4% of the total judgment value to approximately 2.5%. Therefore, the proportion of samples that could not be determined can be reduced from approximately 5% to approximately 3.2%.

### 3.4. Validation of the Origin Discrimination Table

In previous studies that used methods of inorganic content analysis, such as inductively coupled plasma-mass spectrometry or ED-XRF, the reported efficiency was 91.0–94.0%. Moreover, various statistical techniques were applied based on the concentration of 4–8 types of inorganic compounds [19,20,21]. Lee et al. [5] utilized FT-NIRS, a method of organic content analysis. The reported efficiency was 96.1–96.5% based on the difference in the absorbance spectra of organic compounds using the NIR. Through the gene-based analysis in this study, 595 out of 630 domestic samples were predicted as being domestic, with 94.4% sensitivity. In addition, 446 out of 466 imported samples were predicted as being foreign, with 95.7% selectivity. Finally, the efficiency of the origin discrimination table was 95.0%. Conversely, in the analysis based on 10 markers (marker 1–10), 506 of 630 domestic samples were predicted to be domestic, achieving a sensitivity of 80.3%. In addition, 411 of 466 imported samples were predicted to be foreign, demonstrating a selectivity of 88.2%. However, the overall efficiency of the origin discrimination table was 83.7%, and a significant reduction was observed when compared to the analysis with 11 markers. Thus, the analytical method developed in this study exhibited a similar level of efficiency to conventional physicochemical analyses (95.0% vs. 91.0–96.5%) (Table 5).

To determine the practicality of the origin discrimination table proposed in this study, soybean samples from 2019 to 2022, with accurately identified origins, were collected, and their judgment values were applied to the discrimination table.

The judgment values calculated using the 11 InDel markers selected for 30 domestic and 30 imported soybeans were applied to the discrimination table. As shown in Table 6, 29 out of 30 domestic soybeans were discriminated as domestic, showing a 96.7% domestic predictive rate. Furthermore, all 30 imported soybeans were discriminated as foreign, demonstrating a 100.0% foreign predictive rate. Substantially high levels of domestic and foreign predictive rates were obtained at 98.3% on average for the discrimination table using 11 gene-based markers. These results suggest that the discrimination table can effectively discriminate between domestic and imported soybean varieties despite the slightly low level of domestic soybean discrimination caused by a low sensitivity (94.4%) and domestic predictive rate (96.7%) compared to the selectivity (95.7%) and foreign predictive rate (100.0%).

The conventional physicochemical analyses of organic and inorganic compounds for soybean origin discrimination are classification methods for variation in cultivation conditions using statistical techniques. As such, the predictive rate and efficiency may vary [22,23,24]. By contrast, gene-based analyses are independent of compositional changes depending on the cultivation conditions. Soybeans that have been developed to adapt to the climate and pests of each country are known to be unable to be grown successfully in other countries. In particular, the production areas of soybeans imported into Korea are limited to some areas, and these areas have different latitudes and longitudes, compared to Korea. Therefore, it is highly unlikely that imported soybeans can be grown domestically. Therefore, it has been possible to accurately determine the country of origin by utilizing the unique genetic characteristics of soybean varieties.

## 4. Conclusions

In Korea, the price difference of soybeans is 3–5 times more or less than other countries, depending on the soybean origin. Due to the large price difference between domestic and imported soybeans, there is a good possibility that sellers will misrepresent the country of origin of the soybeans. Currently, the origin of soybeans is identified using physicochemical analysis methods such as NIR and XRF to prevent origin misrepresentation; however, because existing physicochemical methods involve the crushing and testing of large amounts of samples, there is a limitation to the extent to which the country of origin can be identified when domestic and imported soybeans are mixed. Consequently, sellers are taking advantage of this limitation and selling a mixture of soybeans from different origins. Therefore, we developed a gene-based analysis method that can identify the country of origin of soybeans on a grain-by-grain basis. In summary, the country-of-origin identification method developed with 11 InDel markers showed an efficiency of 95%, and the validation process of the country-of-origin identification table showed a prediction rate of 98.3%, confirming that the country-of-origin identification at the grain level has a high accuracy. Based on these results, the method developed in this study can be applied to identify the origin of soybeans, and, if combined with existing physicochemical methods, it is expected to prevent illegal acts including the misrepresentation of origin with a higher accuracy.

Furthermore, genetically modified soybeans with herbicide resistance are currently cultivated in several countries, and the use of herbicides, such as glyphosate, saflufenacil, and carfentrazone-ethyl, is rapidly increasing. Conversely, in Korea, the cultivation of genetically modified soybeans is prohibited, and the unintentional tolerance level is maintained below 3%. Therefore, by confirming the country of origin, it is possible to prevent the domestic distribution of genetically modified soybeans and mitigate exposure to harmful substances, such as herbicides [25,26].

Moreover, the genetics-based method for determining the origin of soybeans developed in this study can be applied in quality management during the food manufacturing process. In particular, it is expected to be applicable to intermediate stages (meju) or final products (soybean paste, natto, doenjang, gochujang, etc.) of fermented foods using soybeans.

## Figures and Tables

**Table 1:** A list of the eleven allele-specific PCR markers used for the origin discrimination of soybean in this study.

Set	Marker	Final Primer Conc. (ng/μL)	Forward Sequence	Reverse Sequence	Size (bp)
A	1	1.05	CCAATATCGATCTTTACCAATTCA	TAGCATATTAATCAAACATTTTCTAAA	102
B	3	0.09	TTGGACTGGAGCGTGGAGC	CACCCAAATGGTCATTAGCC	473
	4	0.35	CAGAGCTTCAGTTCTTGACATCAG	TCAAAGAAACAAAACATAGGAAGATG	138
	11	1.05	CGAAATTTTGAAATATACTTGAGAGGA	GCAGGTTCTCATGCAAAATG	112
C	2	0.18	TGAGTGGGTGTGTGTAATAAGTCTT	TGATGGGTTGGACGGTCTAT	238
	5	1.05	CACCCACTCGTTTATCTCGTC	GCGTGTTTGGACTTGGATTG	107
D	6	1.05	TGTATTTGGGACAACTTATTACGTG	CGCACATTAAACACATGTGAAC	459
	7	0.35	CCTTGGTCTTCCACTGCG	TTCGTATTGGGGGTTCAAAA	246
	8	0.35	GGGCATGTCGTCAAGCTTGTC	CCACCTACCCGCAAAACGAT	112
E	9	0.18	TTCTTTCGAGTATTCCCTTTCG	TAGGTGCCTTACGAAAGTTATTATAA	324
F	10	0.35	GCGAATCCAAGACCTAAGTCAG	AAACCACTTGGGTGCCTTTA	872

**Table 2:** Assignment of judgment values for 16 standard samples using 11 InDel markers.

No.	Marker Name	Marker 1	Marker 2	Marker 3	Marker 4	Marker 5	Marker 6	Marker 7	Marker 8	Marker 9	Marker 10	Marker 11	Judgment Value
	**Score**	**1**	**2**	**4**	**8**	**16**	**32**	**64**	**128**	**256**	**512**	**1024**	
1	Daewon	1	2	4		16			128		512	1024	1687
2	Taekwang	1				16				256	512		785
3	Pungsannamul	1	2	4	8	16	32		128			1024	1215
4	Seonyu *	1			8	16			128		512		665
5	Daepung	1	2	4	8		32	64			512		623
6	Sinhwa	1		4	8	16	32		128				189
7	Hwangkeum	1	2	4		16	32		128		512	1024	1719
8	Nampung	1	2		8	16	32			256	512		827
9	Cheonsang	1	2			16	32			256	512		819
10	Uram	1	2		8	16					512	1024	1563
11	Hwangkeumol *	1			8	16			128		512		665
12	Saedanbaek	1		4	8				128		512	1024	1677
13	Pungwon	1		4		16	32	64		256	512	1024	1909
14	Cheongja1	1	2			16	32		128			1024	1203
15	Cheongja3	1	2			16				256	512		787
16	Williams82	1	2	4	8	16	32	64	128	256	512	1024	2047

* Standard samples with overlapping judgment values.

**Table 3:** Discrimination table of domestic soybean using judgment values of 11 markers for soybean origin.

No.	Judgment Value	Marker 1	Marker 2	Marker 3	Marker 4	Marker 5	Marker 6	Marker 7	Marker 8	Marker 9	Marker 10	Marker 11	Duplicate Sample	Note
		1	2	4	8	16	32	64	128	256	512	1024		
1	25	1			8	16							9	
2	31	1	2	4	8	16							1	
3	55	1	2	4		16	32						6	
4	89	1			8	16		64					8	
5	151	1	2	4		16			128				14	
6	157	1		4	8	16			128				6	
7	189	1		4	8	16	32		128				12	Sinhwa
8	409	1			8	16			128	256			4	
9	425	1			8		32		128	256			1	
10	529	1				16					512		45	Taekwang
11	537	1			8	16					512		14	
12	659	1	2			16			128		512		5	
13	663	1	2	4		16			128		512		8	
14	665	1			8	16			128		512		10	Hwangkeumol /Seonyu
15	667	1	2		8	16			128		512		6	
16	669	1		4	8	16			128		512		8	
17	671 *	1	2	4	8	16			128		512		10	
18	785	1				16				256	512		4	
19	787	1	2			16				256	512		8	Cheongja-3
20	819	1	2			16	32			256	512		27	Cheonsang
21	827	1	2		8	16	32			256	512		16	Nampung
22	879	1	2	4	8		32	64		256	512		21	Daepung
23	937	1			8		32		128	256	512		11	
24	939	1	2		8		32		128	256	512		16	
25	1041	1				16						1024	5	
26	1045	1		4		16						1024	4	
27	1047	1	2	4		16						1024	7	
28	1049	1			8	16						1024	18	
29	1077	1		4		16	32					1024	4	
30	1079	1	2	4		16	32					1024	1	
31	1171	1	2			16			128			1024	6	
32	1175	1	2	4		16			128			1024	24	
33	1183 *	1	2	4	8	16			128			1024	8	
34	1203	1	2			16	32		128			1024	3	Cheongja1
35	1207	1	2	4		16	32		128			1024	8	
36	1215 *	1	2	4	8	16	32		128			1024	11	Pungsannamul
37	1339	1	2		8	16	32			256		1024	6	
38	1427	1	2			16			128	256		1024	4	
39	1431	1	2	4		16			128	256		1024	16	
40	1459	1	2			16	32		128	256		1024	2	
41	1467	1	2		8	16	32		128	256		1024	4	
42	1471	1	2	4	8	16	32		128	256		1024	74	
43	1563	1	2		8	16					512	1024	13	Uram
44	1567	1	2	4	8	16					512	1024	1	
45	1595	1	2		8	16	32				512	1024	6	
46	1677	1		4	8				128		512	1024	32	Saedanbaek
47	1681	1				16			128		512	1024	7	
48	1683	1	2			16			128		512	1024	1	
49	1687	1	2	4		16			128		512	1024	58	Daewon
50	1695 *	1	2	4	8	16			128		512	1024	6	
51	1719	1	2	4		16	32		128		512	1024	14	Hwangkeum
52	1909	1		4		16	32	64		256	512	1024	16	Pungwon
53	1939	1	2			16			128	256	512	1024	1	

* Overlapping judgment values for domestic and foreign soybean varieties.

**Table 4:** Discrimination table of imported soybeans using judgment values of 11 markers for soybean origin.

No.	Judgment Value	Marker 1	Marker 2	Marker 3	Marker 4	Marker 5	Marker 6	Marker 7	Marker 8	Marker 9	Marker 10	Marker 11	Duplicate Sample	Note
		1	2	4	8	16	32	64	128	256	512	1024		
1	245	1		4		16	32	64	128				3	
2	247	1	2	4		16	32	64	128				11	
3	253	1		4	8	16	32	64	128				5	
4	429	1		4	8		32		128	256			2	
5	447	1	2	4	8	16	32		128	256			8	
6	493	1		4	8		32	64	128	256			6	
7	495	1	2	4	8		32	64	128	256			12	
8	541	1		4	8	16					512		2	
9	573	1		4	8	16	32				512		1	
10	601	1			8	16		64			512		3	
11	671 *	1	2	4	8	16			128		512		4	
12	701	1		4	8	16	32		128		512		1	
13	703	1	2	4	8	16	32		128		512		1	
14	733	1		4	8	16		64	128		512		2	
15	735	1	2	4	8	16		64	128		512		1	
16	743	1	2	4			32	64	128		512		4	
17	751	1	2	4	8		32	64	128		512		8	
18	757	1		4		16	32	64	128		512		1	
19	765	1		4	8	16	32	64	128		512		6	
20	767	1	2	4	8	16	32	64	128		512		4	
21	943	1	2	4	8		32		128	256	512		6	
22	1023	1	2	4	8	16	32	64	128	256	512		11	
23	1055	1	2	4	8	16						1024	8	
24	1059	1	2				32					1024	1	
25	1085	1		4	8	16	32					1024	12	
26	1165	1		4	8				128			1024	7	
27	1179	1	2		8	16			128			1024	21	
28	1181	1		4	8	16			128			1024	8	
29	1183 *	1	2	4	8	16			128			1024	6	
30	1197	1		4	8		32		128			1024	16	
31	1215*	1	2	4	8	16	32		128			1024	9	
32	1229	1		4	8			64	128			1024	4	
33	1261	1		4	8		32	64	128			1024	10	
34	1263	1	2	4	8		32	64	128			1024	7	
35	1277	1		4	8	16	32	64	128			1024	5	
36	1279	1	2	4	8	16	32	64	128			1024	12	
37	1323	1	2		8		32			256		1024	2	
38	1343	1	2	4	8	16	32			256		1024	9	
39	1387	1	2		8		32	64		256		1024	14	
40	1407	1	2	4	8	16	32	64		256		1024	8	
41	1487	1	2	4	8			64	128	256		1024	18	
42	1535	1	2	4	8	16	32	64	128	256		1024	4	
43	1565	1		4	8	16					512	1024	14	
44	1575	1	2	4			32				512	1024	12	
45	1597	1		4	8	16	32				512	1024	5	
46	1599	1	2	4	8	16	32				512	1024	1	
47	1647	1	2	4	8		32	64			512	1024	4	
48	1691	1	2		8	16			128		512	1024	3	
49	1693	1		4	8	16			128		512	1024	8	
50	1695 *	1	2	4	8	16			128		512	1024	1	
51	1709	1		4	8		32		128		512	1024	4	
52	1711	1	2	4	8		32		128		512	1024	7	
53	1725	1		4	8	16	32		128		512	1024	10	
54	1727	1	2	4	8	16	32		128		512	1024	13	
55	1741	1		4	8			64	128		512	1024	8	
56	1757	1		4	8	16		64	128		512	1024	14	
57	1759	1	2	4	8	16		64	128		512	1024	13	
58	1773	1		4	8		32	64	128		512	1024	19	
59	1775	1	2	4	8		32	64	128		512	1024	2	
60	1791	1	2	4	8	16	32	64	128		512	1024	4	
61	1789	1		4	8	16	32	64	128		512	1024	1	
62	1951	1	2	4	8	16			128	256	512	1024	7	
63	1965	1		4	8		32		128	256	512	1024	8	
64	1967	1	2	4	8		32		128	256	512	1024	13	
65	1981	1		4	8	16	32		128	256	512	1024	4	
66	1983	1	2	4	8	16	32		128	256	512	1024	4	
67	2015	1	2	4	8	16		64	128	256	512	1024	2	
68	2031	1	2	4	8		32	64	128	256	512	1024	1	
69	2045	1		4	8	16	32	64	128	256	512	1024	1	
70	2047	1	2	4	8	16	32	64	128	256	512	1024	0	Williams82

* Overlapping judgment values for domestic and foreign soybean varieties.

**Table 5:** Classification performance parameters of gene-based analysis for discriminating Korean and imported soybeans.

Statistical Value		No. of Sample	
Classification	TD (True Domestic Product)	595	-
	FD (False Domestic Product)	35	-
	TF (True Foreign Product)	-	446
	FF (False Foreign Product)	-	20
	Total	630	466
	Sensitivity	$=(\frac{595}{595\;+\;35})\times 100=94.4\%$	-
	Selectivity	-	$=(\frac{446}{466\;+\;20})\times 100=95.7\%$
	Efficiency	$=(\frac{595\;+\;446}{595\;+\;35\;+\;446\;+\;20})\times 100=95.0\%$	

**Table 6:** Validation results for the discrimination table using judgment values of 11 markers for soybeans.

Classification	No. of Samples			Predictive Rate
	Total	Domestic	Foreign	
Total	60	30	30	$=\frac{59}{60}\times 100=98.3\%$
Domestic	30	29	1	$=\frac{29}{30}\times 100=96.7\%$
Imported	30	0	30	$=\frac{30}{30}\times 100=100.0\%$

## Data Availability

Data is contained within the article.

## References

[1] Kudełka W., Kowalska M., Popis M. (2021). Quality of soybean products in terms of essential amino acids composition. Molecules.

[2] Trade Statistics. https://stat.kita.net.

[3] NAQS (National Agricultural Products Quality Management Service) (2022). 2022 Annual Report. 11-1543145-000037-10.

[4] Lee J.-H., Kang D.-J., Jang E.-H., Hur S.-H., Shin B.-K., Han G.-T., Lee S.-H. (2020). Discrimination of geographical origin for soybean using ED-XRF. Korean J. Food Sci. Technol..

[5] Lee J.H., An J.M., Kim H.J., Shin H.C., Hur S.H., Lee S.H. (2022). Rapid discrimination of the country origin of soybeans based on FT-NIR spectroscopy and data expansion. Food Anal. Methods.

[6] Ahn H.-G., Kim Y.-H. (2012). Discrimination of Korean domestic and foreign soybeans using near infrared reflectance spectroscopy. Korean J. Crop Sci..

[7] Agarwal M., Shrivastava N., Padh H. (2008). Advances in molecular marker techniques and their applications in plant sciences. Plant Cell Rep..

[8] Sohn H.B., Kim S.J., Hwang T.Y., Park H.M., Lee Y.Y., Markkandan K., Lee D., Lee S., Hong S.Y., Song Y.H. (2017). Barcode system for genetic identification of soybean [*Glycine max* (L.) Merrill] cultivars using InDel markers specific to dense variation blocks. Front. Plant Sci..

[9] Kwon Y. (2016). DNA Fingerprinting analysis for soybean (*Glycine max*) varieties in Korea using a core set of microsatellite marker. J. Plant Biotechnol..

[10] Tripathi M.K., Tripathi N., Tiwari S., Mishra N., Sharma A., Tiwari S., Singh S. (2023). Identification of Indian soybean (*Glycine max* [L.] Merr.) genotypes for drought tolerance and genetic diversity analysis using SSR markers. Scientist.

[11] Hou X., Li L., Peng Z., Wei B., Tang S., Ding M., Liu J., Zhang F., Zhao Y., Gu H. (2010). A platform of high-density INDEL/CAPS markers for map-based cloning in Arabidopsis. Plant J..

[12] Hyten D.-L., Choi I.-Y., Song S., Shoemaker R.-C., Nelson R.-L., Costa J.-M., Specht J.-E., Cregan P.-B. (2007). Highly variable patterns of linkage disequilibrium in multiple soybean populations. Genetics.

[13] Chun J., Jin M., Jeong N., Cho C., Seo M.-S., Choi M.-S., Kim D.-Y., Sohn H.-B., Kim Y.-H. (2019). Genetic Identification and Phylogenic Analysis of New Varieties and 149 Korean Cultivars Using 27. InDel markers selected from dense variation blocks in soybean (*Glycine max* (L.) Merrill). Korean J. Plant Res..

[14] Sohn H.-B., Song Y.-H., Kim S.-J., Hong S.-Y., Kim K.-D., Koo B.-C., Kim Y.-H. (2018). Identification and chromosomal reshuffling patterns of soybean cultivars bred in Gangwon-do using 202. InDel markers specific to variation blocks. Korean J. Breed. Sci..

[15] Sohn H.-B., Kim S.-J., Hwang T.-Y., Park H.-M., Lee Y.-Y., Koo B.-C., Kim Y.-H. (2017). Chromosome reshuffling patterns of Korean soybean cultivars using genome-wide 202 InDel markers. Korean J. Breed. Sci..

[16] (2012). NATA Technical Note 17. Guidelines for the Validation and Verification of Quantitative and Qualitative Test Method.

[17] Pomerantsev A.L., Rodionova O.Y. (2021). New trends in qualitative analysis: Performance, optimization, and validation of multi-class and soft models. TrAC Trends Anal. Chem..

[18] Kruglyak L., Nickerson D.A. (2001). Variation is the spice of life. Nat. Genet..

[19] Nguyen-Quang T., Bui-Quang M., Truong-Ngoc M. (2021). Rapid identification of geographical origin of commercial soybean marketed in Vietnam by ICP-MS. J. Anal. Methods Chem..

[20] Otaka A., Hokura A., Nakai I. (2014). Determination of trace elements in soybean by X-ray fluorescence analysis and its application to identification of their production areas. Food Chem..

[21] Kang D.-J., Moon J.-Y., Lee D.-G., Lee S.-H. (2016). Identification of the geographical origin of cheonggukjang by Using Fourier Transform Near-Infrared Spectroscopy and Energy Dispersive X-ray Fluorescence Spectrometry. Korean J. Food Sci. Technol..

[22] Drivelos S.A., Georgiou C.A. (2012). Multi-element and Multi-isotope-ratio analysis to Determine the geographical origin of foods in the European Union. TrAC Trends Anal. Chem..

[23] Liu X., Zhao Y., Qi P., Liu Y., Li X., Deng W., Zhang J., Sadiq F.A., Sang Y., Zhang A. (2022). Origin verification of Chinese concentrated apple juice using stable isotopic and mineral elemental fingerprints coupled with chemometrics. J. Food Compos. Anal..

[24] Schütz D., Riedl J., Achten E., Fischer M. (2022). Fourier-transform near-infrared spectroscopy as a fast screening tool for the verification of the geographical origin of grain maize (*Zea mays* L.). Food Control.

[25] Perry E.-D., Ciliberto F., Hennessy D.-A., Moschini G. (2016). Genetically engineered crops and pesticide use in U.S. maize and soybeans. Sci. Adv..

[26] Won O.-J., Hong S.-Y., Suh E.-J., Park J.-S., Lee H.-S., Park J.-K., Ryu J.-S., Han W.-Y., Han K.-S., Song D.-Y. (2021). Possibility of using non-selective herbicides as desiccants for improving soybean harvest efficiency. Korean J. Crop Sci..

